# Epidermoid Cyst Mimicking a Cystic Parotid Tumor: A Diagnostic Dilemma Deciphered Intraoperatively

**DOI:** 10.7759/cureus.54535

**Published:** 2024-02-20

**Authors:** Raparthi Bhuvan Chandra, Sneha Pendem, Kathiravan Selvarasu, Murugesan Krishnan, Muthusekhar M. R.

**Affiliations:** 1 Oral and Maxillofacial Surgery, Saveetha Dental College and Hospitals, Saveetha Institute of Medical and Technical Sciences, Saveetha University, Chennai, IND

**Keywords:** innovative, novel, diagnosis, surgery, epidermoid cyst

## Abstract

Cystic lesions in the preauricular may have various histological origins, ranging from the skin to the acinar and non-acinar lesions from the parotid. Though advanced radiological investigations provide a good insight into the diagnosis of these lesions, diagnostic dilemmas may still prevail and warrant good clinical and surgical acumen to provide optimal treatment. The aim of the current report is to describe a case of an epidermoid cyst that mimicked a parotid cyst and discuss in detail the probable differential diagnosis and their management strategies.

## Introduction

Epidermoid cysts are developmental, benign, and cutaneous cysts that are commonly found on the scalp, face, and neck, followed by the trunk. In the head and neck region, they occur in the orbital region, buccal and submental areas, junction of the hard and soft palate, and rarely in the labial mucosa. These cysts are benign cystic malformations that are derived from the ectoderm and constitute 1.6-6.9% of all cysts in the head and neck [1,2], with 1.6% occurring in the oral cavity. These cysts are lined by epidermis-like epithelium and develop due to the entrapment of cutaneous epithelium in the underlying mesenchyme during development [3,4].

They can develop in isolation or multifocal form, as in Gardner’s syndrome [4]. Seldom do these cysts originate from infundibular epithelium hyperplasia in response to an inflammatory process in the hair follicle. In these situations, they may present in the latter phases of life and must be differentiated from the common swellings in that region. Traumatic or iatrogenic entrapment of the epidermis may also lead to the development of these cysts in adult patients.

Various other swellings may present with a similar clinical presentation, including parotid duct cysts, cystic lesions of the parotid gland, and intraglandular vascular (hemangiomas) and non-vascular benign tumors. These lesions add to the diagnostic dilemma and will require different surgical approaches for management, including parotidectomy if needed, which can have considerable morbidity. Hence, precise planning is essential for the appropriate management of these lesions [4,5].

The purpose of this paper is to describe a case of a pre-auricular epidermoid cyst that resembled a parotid cyst and to discuss the differential diagnosis and management strategy.

## Case presentation

**Figure 1:**
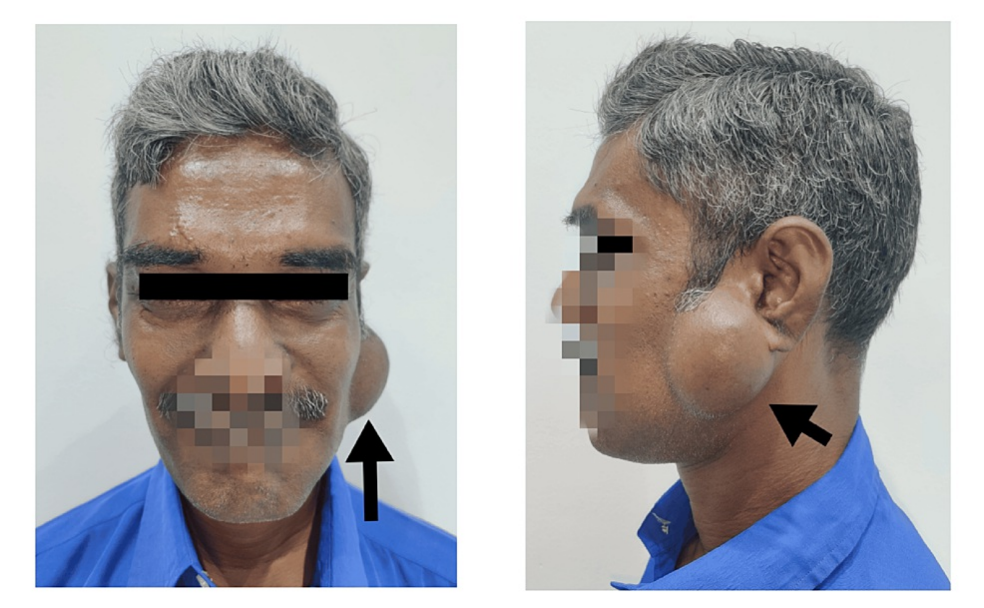
Preoperative extraoral frontal and profile views

A 51-year-old male patient reported to the OPD with a complaint of swelling in the left pre-auricular region of three years duration that grew gradually to the present size. There were no similar swellings elsewhere in the body. Extraoral examination revealed a non-tender, painless swelling in the left preauricular area, measuring about 6 cm × 6 cm × 3 cm, extending from the tragus of the ear to the angle of the mandible, with elevation of the ear lobe (Figure 1).

**Figure 2:**
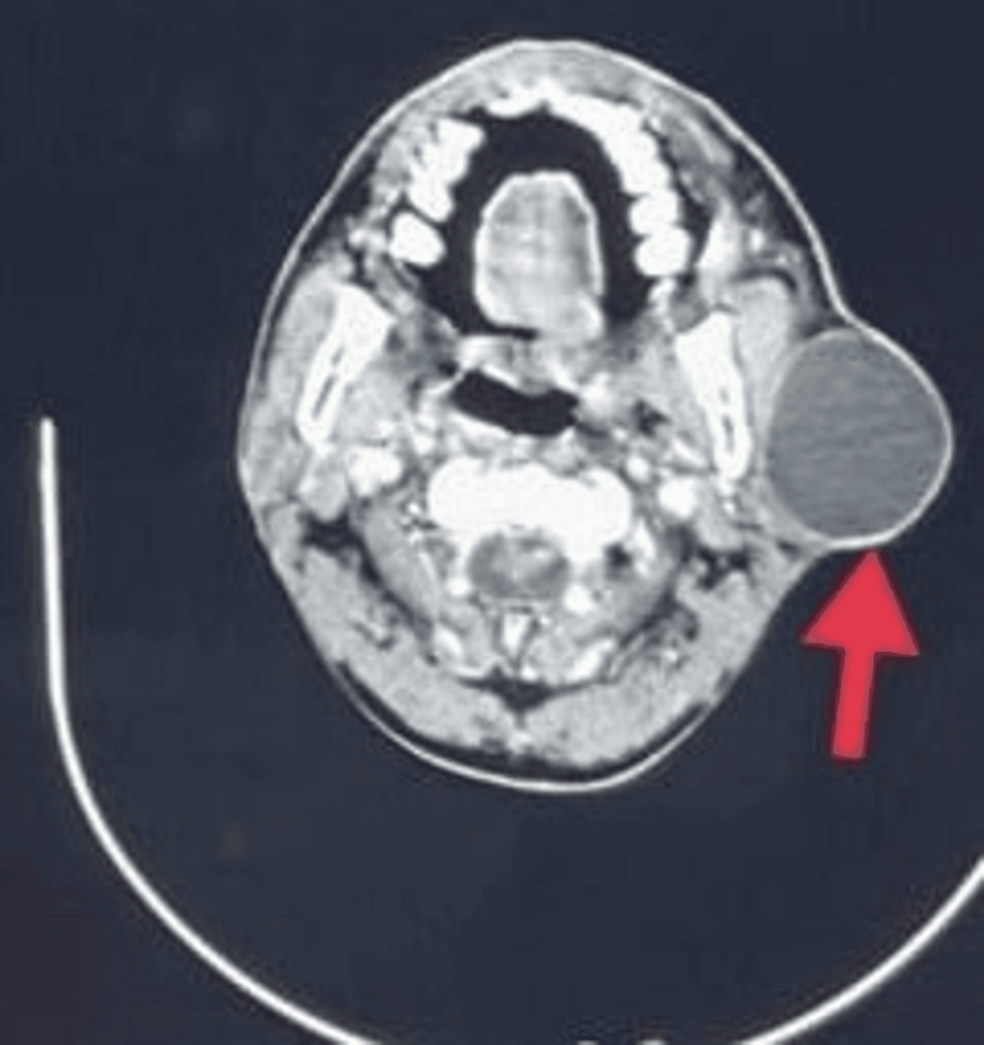
Preoperative CECT scan (axial section) showing defined lesion (6.3 × 5.8 × 4.5 cm) with hyperdense borders (arrow)

On palpation, the swelling is soft in consistency, non-fluctuant, not compressible, and not reducible. The swelling was not freely mobile over the masseter, and the skin was pinchable over the lesion with no visible punctum. Facial nerve function was graded using House-Brackmann grading and was found to be grade I. A provisional diagnosis of cutaneous cystic lesion was arrived at; however, the diagnosis of cystic degeneration of parotid tumors couldn’t be ruled out clinically, and the patient was subjected to further investigations, including a contrast-enhanced computed tomography (Figure 2), which revealed a well-defined thin-walled non-enhancing hypodense cystic lesion with minimal wall enhancement with the contrast of size 6.3 × 5.8 × 4.5 cm at its greatest dimension on axial section.

**Figure 3:**
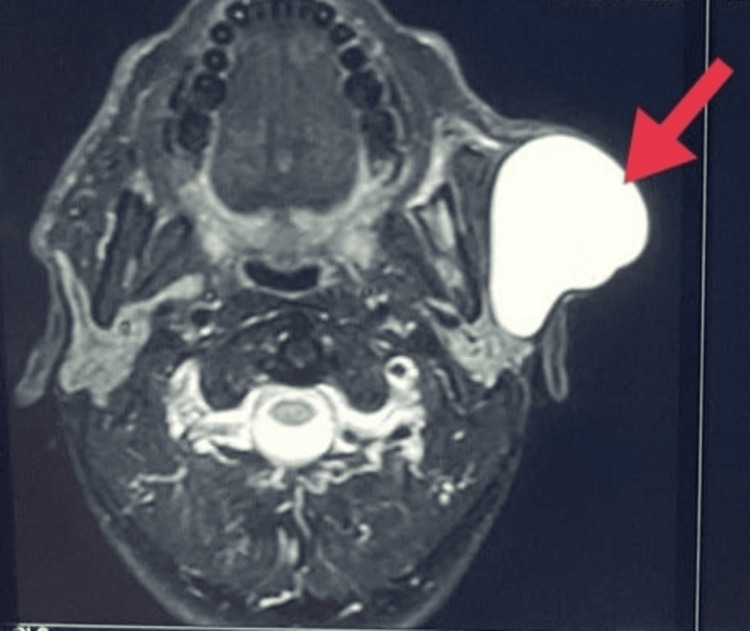
T2-weighted MRI image (axial section) showing a well-defined hyperintense lesion in the subcutaneous plane abutting the parotid gland

T2-weighted magnetic resonance imaging (MRI) revealed a thin-walled, well-defined, hyperresonant cystic lesion in the subcutaneous plane of the preauricular region abutting the parotid gland (Figure 3).

A provisional diagnosis of parotid cyst/cystic degeneration in parotid neoplasm was arrived at, with epidermoid cyst being the differential diagnosis based on the clinical and radiological findings. 

**Figure 4:**
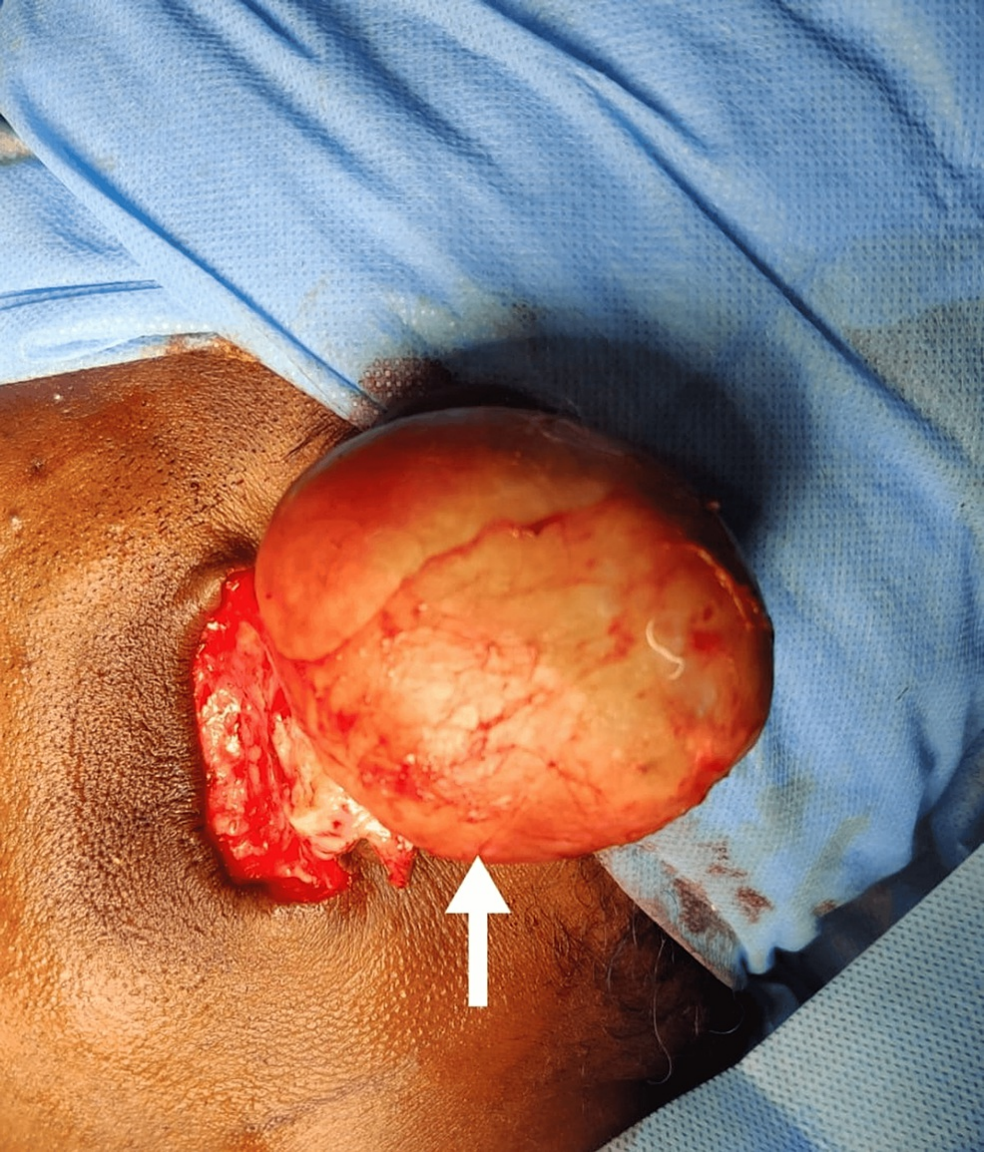
Surgical exposure of the lesion (arrow)

The patient was planned for surgical excision of the same with superficial parotidectomy as part of clearance. The lesion was approached through modified Blair incision markings and was placed with an incision to remove excess skin tissue. Dissection of the lesion was carried out in the subcutaneous tissue plane (Figure 4).

**Figure 5:**
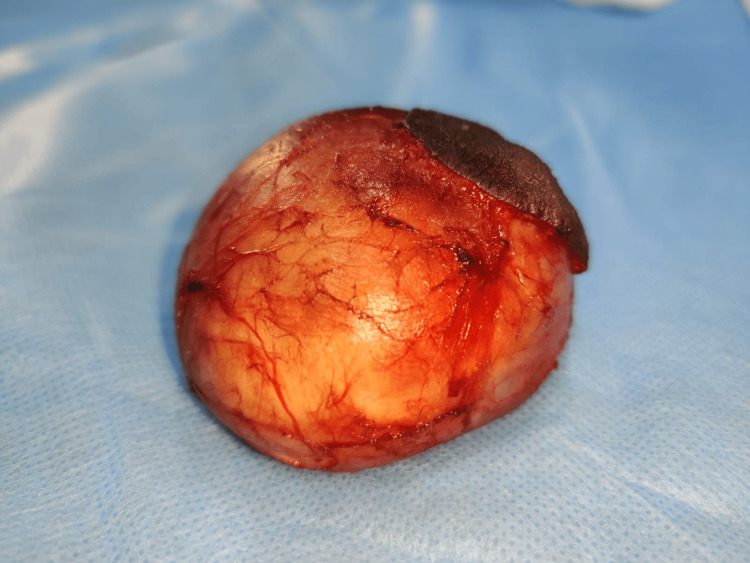
Lesion excised in toto

A surgical plane of dissection was obtained between the lesion and the capsule, which was excised in toto. The specimen was sent for histopathological examination, and layer-wise closure was done (Figure 5).

**Figure 6:**
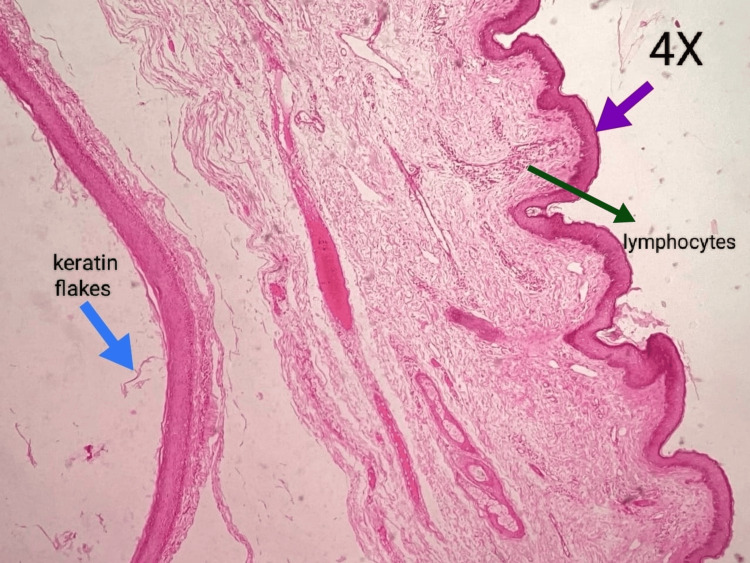
Histopathological photomicrograph (4× magnification)

The cystic lumen revealed white, cheesy, and pungent-smelling fluid. The microscopic section shows the epithelial lining and connective tissue wall. The epithelial lining is ortho-keratinized stratified squamous epithelium with a prominent granular cell layer, and the epithelial connective tissue surface is flat. There is evidence of abundant laminated keratin flakes in the cystic lumen. The supporting fibrous connective tissue wall shows moderate diffuse chronic inflammatory cell infiltration, predominantly lymphocytes, and moderate vascularity. Areas of hemorrhage and adipose tissue are also evident. This histopathology is suggestive of an epidermoid cyst (Figure 6).

**Figure 7:**
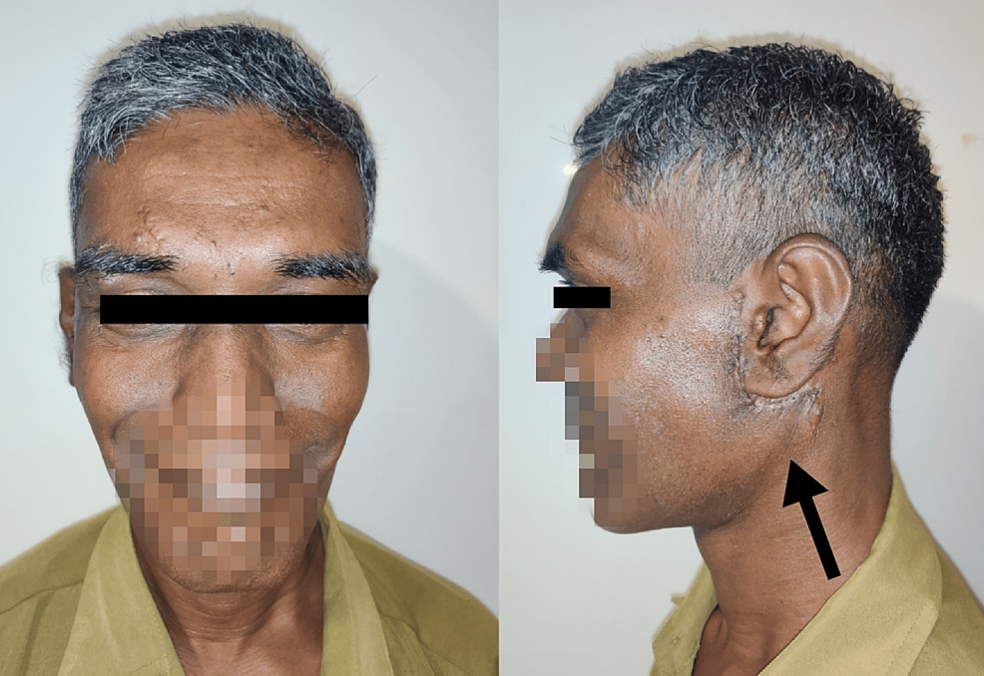
Post-operative pictures of the patient

The patient was reviewed periodically for six months and had no other complaints. Sutures were removed after 15 days, and there was no sign of infection or wound dehiscence, although there was a surgical scar post-operative (Figure 7).

## Discussion

Swellings of the parotid region, though extremely common, are dicey for diagnosis and may range from intrinsic tumors of the parotid gland to benign or malignant neoplasms arising from non-salivary tumors from within the gland or tissues surrounding the same [6]. Though intrinsic glandular tumors of the parotid have classic clinical features, they may be indistinct when these swellings originate from the tail of the parotid gland. These lesions often present a diagnostic dilemma for clinicians and often need thorough radiological evaluation for appropriate diagnosis and treatment planning. Diagnosis may often be confounded when recurrent infections lead to pericapsular fibrosis and the loss of fat planes.

Common intrinsic tumors that arise from the parotid include pleomorphic or monomorphic adenoma malignancies that commonly include mucoepidermoid carcinoma (MEC) and adenoid cystic carcinoma. These tumors, especially MEC, may undergo cystic degeneration and present in the aforesaid form, leading to a diagnostic dilemma. Conventional investigative modalities provide an accurate diagnosis, but extreme degrees of cystic degeneration may interfere with the diagnosis. In these cases, FNAC is often not diagnostic and may lead to tumor seeding; hence, it is not often recommended. This was the primary factor confounding the diagnosis in our case [7]. In our case, as the swelling is huge and fluid-filled, we did not opt for an FNAC preoperatively, as it can interfere with the plane of dissection, which can be troublesome intraoperatively. Apart from this, FNAC for parotid malignancies with cystic degeneration can lead to tumor seeding, contributing to tumor recurrence. On the table, FNAC was performed, with care to include the site of FNAC in the cutaneous excision, which revealed a pungent, cheesy white liquid and was diagnostically inconclusive.

Non-acinar tumors include those arising from vascular, lymphatic, or neural structures, like hemangiomas, lymphomas, and neurofibromas. Our case is different from that of hemangioma, as it did not show contrast enhancement on CECT and clearly revealed it to be a fluid-filled cavity, also aiding in ruling out solid tumors of neural or lymphatic origin [8]. Warthin’s tumor is another parotid lesion with a similar clinical presentation; however, it is usually bilateral and presents with a chocolate-colored fluid aspirate. There is an eosinophilic coagulum present within the cystic spaces, which accounts for this color aspirate. CECT reveals modular mass with cystic spaces and moderate contrast enhancement, unlike epidermoid cysts.

Lymphomas of non-nodal origin show rapid growth and can be ruled out from epidermoid cysts, which reveal a progressive slow growth pattern ranging from six months to two years [9,10]. The majority of parotid lymphomas are non-Hodgkin-type and B-cell-type. Medical conditions associated with lymphomas of the salivary gland include mumps, benign lymphoepithelial lesions (Mikulicz disease), and Sjogren’s syndrome [11]. Most of them appear to be painless, rapidly growing swellings of the parotid gland and are often managed by chemotherapy drugs like cyclophosphamide, vincristine, and doxorubicin. 

Kaposi’s sarcoma is another lesion that may be present in this age group (50-70 years). It is a solid tumor of vascular endothelial origin that reveals a rapid growth pattern and is common in patients with HIV infection. CECT reveals a nodular mass with irregular borders and infiltration of the adjacent tissues with contrast uptake. Radiation therapy and chemotherapy with intralesional vinblastine are proven as good choices of treatment for Kaposi’s sarcoma [12,13]. Negative serology (HIV-ELISA) and a lack of contrast enhancement helped in ruling out Kaposi’s tumors in our patient.

Another cutaneous lesion that is common in the hair-bearing area is the sebaceous cyst. This is usually caused by an obstruction of the sebaceous duct at the base of the hair follicle. This lesion classically presents with a punctum. The classic clinical presentation of these lesions involves the non-pinchability of the skin and their mobility over the underlying masseter during clenching, which was positive in our case. These lesions present as hypoechoic or hypodense lesions on radiology and do not show any contrast enhancement similar to parotid cysts. A lesion in the subcutaneous plane that does not encroach on the parotid tissue in the radiograph (either CECT or MRI) will be a diagnostic guideline to differentiate between cystic parotid lesions and epidermoid cysts.

Epidermoid or dermoid cysts are extremely rare, are usually seen along the lines of embryonic lines of fusion, and are often congenital [14,15]. They usually occur due to the entrapment of the cutaneous epithelium into the underlying connective tissue. Though these lesions are congenital in nature, they may be caused by accidental entrapment of the epidermis secondary to trauma. This probably contributed to the incidence of epidermoid cysts in our patient.

In the current scenario, there was no distinct punctum over the swelling, and the swelling was not freely mobile over the underlying masseter. This could be attributed to the fibrosis caused between the cyst lining and the underlying parotid capsule secondary to a recurrent cutaneous infection. The provisional diagnosis was based on the clinical demographics and radiological presentation. Though preoperative imaging provided insight into the lesion being non-vascular, the probability of cystic degeneration from the parotid malignancy could not be ruled out [16-21]. This emphasizes the need to plan the management strategy and to be prepared for radical procedures, including parotidectomy if needed, as part of clearance in case of untoward intraoperative findings. These lesions need histological evaluation to prevent misdiagnosis and inadequate treatment, as malignancies such as MEC are often aggressive and necessitate superficial or total parotidectomy with neck dissection to address cases of cervical metastasis.

## Conclusions

To conclude, cutaneous cystic lesions, including epidermoid cysts, can often be misdiagnosed as glandular lesions from the parotid. Seldom malignancies, including MEC, may mimic these cutaneous malignancies, like cutaneous squamous cell carcinoma of the parotid, and will have to be diagnosed accurately to deliver appropriate treatment. Pre-operative imaging, though it provides clues for diagnosis, sometimes may not be adequate and needs to be supplemented with a good histopathological exam, including a frozen section. This aids in delivering the best possible treatment to the patient, minimizing morbidity and recurrence.

## References

[1] Boricha V, Shetty N, Vachhani D, Bangera D, Surabhi V (2018). Just another sebaceous cyst?? An epidermoid cyst of head and neck region: a case report. Austin J Dent.

[2] Baisakhiya N, Deshmukh P (2011). Unusual sites of epidermoid cyst. Indian J Otolaryngol Head Neck Surg.

[3] Dutta M, Saha J, Biswas G, Chattopadhyay S, Sen I, Sinha R (2013). Epidermoid cysts in head and neck: our experiences, with review of literature. Indian J Otolaryngol Head Neck Surg.

[4] Mevorah B, Bovet R (1984). Treatment of retroauricular keratinous cysts. J Dermatol Surg Oncol.

[5] Hegde PN, Prasad H L K, Kumar Y S, Sajitha K, Roy PS, Raju M, Shetty V (2013). A rare case of an epidermoid cyst in the parotid gland - which was diagnosed by fine needle aspiration cytology. J Clin Diagn Res.

[6] Ozer E, Kanlikama M, Bayazit YA, Mumbuç S, Sari I, Gök A (2003). A unique case of an epidermoid cyst of the pterygopalatine fossa and its management. Int J Pediatr Otorhinolaryngol.

[7] Supriya M, Denholm S, Palmer T (2008). Seeding of tumor cells after fine needle aspiration cytology in benign parotid tumor: a case report and literature review. Laryngoscope.

[8] Ravindranath AP, Ramalingam K, Natesan A, Ramani P, Premkumar P, Thiruvengadam C (2009). Epidermoid cysts: an exclusive palatal presentation and a case series. Int J Dermatol.

[9] Asad Ullah M, Ahmed A, Hyder SM, Javed K, Naeem MQ (2023). An unusual case of sublingual epidermoid cyst mimicking plunging ranula. Cureus.

[10] Janarthanam J, Mahadevan S (2012). Epidermoid cyst of submandibular region. J Oral Maxillofac Pathol.

[11] Andola SK, Masgal MM, Reddy RM (2016). Diffuse large B-cell lymphoma of the parotid gland: cytological, histopathological, and immunohistochemical features: A rare case report. J Cytol.

[12] Quéro L, Palich R, Valantin MA, On Behalf Of Cancervih Working Group (2022). The role of radiotherapy in treating Kaposi’s sarcoma in HIV infected patients. Cancers (Basel).

[13] Boudreaux AA, Smith LL, Cosby CD, Bason MM, Tappero JW, Berger TG (1993). Intralesional vinblastine for cutaneous Kaposi's sarcoma associated with acquired immunodeficiency syndrome. A clinical trial to evaluate efficacy and discomfort associated with infection. J Am Acad Dermatol.

[14] Puranik SR, Puranik RS, Prakash S, Bimba M (2016). Epidermoid cyst: report of two cases. J Oral Maxillofac Pathol.

[15] Bitar MA, Kumar S (2003). Plunging congenital epidermoid cyst of the oral cavity. Eur Arch Otorhinolaryngol.

[16] Mukunyadzi P (2002). Review of fine-needle aspiration cytology of salivary gland neoplasms, with emphasis on differential diagnosis. Am J Clin Pathol.

[17] Yanai Y, Tsuji R, Ohmori S, Tatara N, Kubota S, Nagashima C (1985). Malignant change in an intradiploic epidermoid: report of a case and review of the literature. Neurosurgery.

[18] Weinberg S, Kryshtalskyj B (1995). Epidermoid cyst in a temporomandibular joint dermal graft: report of a case and review of the literature. J Oral Maxillofac Surg.

[19] Zachariades N, Skoura-Kafoussia C (1990). A life-threatening epidermoid cyst of the floor of the mouth: report of a case. J Oral Maxillofac Surg.

[20] Ramakrishnan DS, Abdul Wahab PU, Dhasarathan P, Madhulaxmi M, Kandamani J (2020). Surgical ciliated cyst of the left maxilla-a case report of unusual pathogenesis. Ann Maxillofac Surg.

[21] Rai AK, Singh S, Basu A, Prasad BK (2023). Sublingual epidermoid cyst with cervical lymphadenopathy: a rare case with review of literature. Indian J Otolaryngol Head Neck Surg.

